# Q-S laser micro-drilling and multipass full-beam Q-S laser for tattoo removal — a case series

**DOI:** 10.1007/s10103-021-03431-w

**Published:** 2021-10-04

**Authors:** Leonardo Marini, Susanna Marini, James Cutlan, Irena Hreljac

**Affiliations:** 1 SDC The Skin Doctors Center, via dei Bonomo 5/a, 34126 Trieste, Italy; 2 University Hospital of Wales, Cardiff, Wales UK; 3 Royal Glamorgan Hospital, Llantrisant, Wales UK; 4 Laser and Health Academy, Ljubljana, Slovenia

**Keywords:** Tattoo, Q-S laser, Ablative fractional laser, Combination treatment

## Abstract

The purpose of this study was to evaluate the safety and efficacy of a new combined method of Q-S laser-assisted tattoo removal. Ten patients with 13 professional, mostly mono-chromatic black tattoos were recruited. All tattoos received the same Q-S laser treatment sequence. An objective evaluation of tattoo clearing was assessed by careful analysis of a standardized collection of digital images taken from each tattoo, 2 months after each laser session, with the help of a custom-made pigment-fading percentage photographic ruler. The percentages of pigment clearance and side effects were evaluated by 4 independent dermatologists. Patient satisfaction and perceived discomfort during and post-procedure were evaluated according to specific scales. Clinical evaluators confirmed an average photographic pigment clearance of 97% after a median 4.85 treatment sessions. The Frac-Tat® method required 40% fewer sessions compared to those calculated by Kirby-Desai estimates. Photographic assessment of laser-exposed skin quality performed 2 months after tattoo clearing was considered almost comparable with untreated peripheral skin, confirming a very low side effect score. The Frac-Tat QS laser-assisted tattoo removal sequence used in our study showed a high degree of safety and efficiency, clearing exogenous pigments in a relatively few number of sessions. Preliminary ablative photo-acoustic fractional 1064-nm Q-S laser micro-drilling was considered an essential step in optimizing tattoo removal, increasing wavelength-independent micro-columnar clearing of deeper dermal exogenous pigments. Our preliminary observations also confirmed a significant improvement of tattoo procedure-induced micro-textural changes thanks to a tissue remodeling effect induced by the 1064-nm Q-S fractional laser photo-acoustic ablation.

## Introduction

Tattoos have been well established visual expression means of communication used by single or groups of individuals since ancient times [1, 2]. Skin tattoos are on the rise in modern societies with a parallel increase in tattoo removal requests [3]. The significant rise in tattoo removal procedures was undisputedly triggered by the advent of modern pigment-specific Q-switched (Q-S) laser technologies. Their ultrashort nanosecond (ns) or picosecond (ps) pulses are able to selectively interact with intradermal pigment particles in full accordance with the universally accepted selective photo-thermolysis theory leading to a super-confined photo-thermal-photo-acoustic destruction of wavelength-specific absorbing pigments with minimal tissue collateral damage [4, 5]. Due to these highly specific effects, tissue recovery is relatively short and possible complications and side effects extremely reduced when laser parameters are properly selected [6].

Modern Q-S laser resonators are therefore able to produce micro-concentrations of extremely high peak-power energies confined within ultra-short amounts of time [7–9]. Exposed tissues respond with a unique photo-acoustic reaction associated with plasma formation when wavelength-specific absorbing chromophores are targeted resulting is their ultra-structural fragmentation and partial loss of their wave-specific light absorption properties. A typical immediate short-lived Q-S laser tattoo removal side effect consists of a superficial whitish discoloration described as “pop-corn” effect. It consists of sub-epidermal gas bubbles generated when plasma is produced within pigment-containing cells. These bubbles usually last 20 min, acting as a temporary “optical shield” preventing further laser light penetration when multi-pass strategies are selected. Original Q-S laser-assisted tattoo removal procedures consisted mostly of single laser pass-per-session (PPS) approaches requiring a relatively high number of sessions to obtain optimal pigment clearing. Independently from Q-S laser-assisted tattoo removal strategies, inter-session intervals vary according to the anatomical location of exogenous pigments, ranging from a minimum of 2 months for the upper body to a maximum of three/four for lower limbs. Single laser PPS strategies are therefore long and costly for patients. To try to overcome this problem, Kosida and colleagues proposed the so-called R-20 method consisting in four sequential Q-S laser passes, spaced 20-min apart, during the same laser session. They stated their method was able to significantly reduce the total number of sessions producing better clinical results when compared to single laser PPS techniques [10]. These authors undisputedly paved the way for other multi-layer laser-assisted tattoo removal strategies, even if their method did not come without disadvantages. Treatment sessions were extremely long, considering a minimum of 1-h (20 min × 3) waiting time to be added to the amount of time necessary to perform four full-beam laser passes. Larger tattoos required even more than 2 h per session, putting laser operators and patients under significant pressure. Topical perfluorodecalin (PFD) sheets have been subsequently introduced to shorten inter-laser pass intervals, thanks to their capability of absorbing Q-S laser-induced sub-epidermal gasses. But even this solution did not come free of draw-backs such as increased costs to be inevitably added to laser procedures [11, 12].

**Fig. 1:**
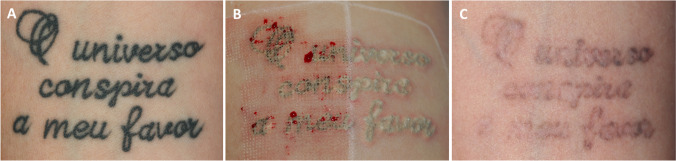
Example of a split-tattoo treatment performed prior to this study. Preliminary micro-drilling performed with a 2940-nm Er:YAG laser in ablative fractional mode immediately before two 1064-nm Nd:YAG full beam laser passes spaced 20-min apart on the left half of tattoo. Two 1064-nm Q-S full beam passes spaced 20-min apart on the right side. **A** Pre-treatment virgin tattoo; **B** after 2 passes of Q-S 1064-nm Nd:YAG laser, left side pre-treated by 2940-nm Er:YAG laser ablative fractional micro-drilling mode; right half treated by two full beam Q-S 1064-nm Nd:YAG laser passes only — note the difference between sub-epidermal gas retaining skin properties after preliminary ablative laser micro-drilling (left) and without micro-drilling (right); **C** 4 months after 2 treatments — left half showing markedly better clearance. Complete clearance was achieved after 5 sessions using the Frac-Tat technique on both sides

Micro-drilling of epidermal layers by fractional-mode 2940-nm Er:YAG or CO^2^ lasers performed prior to Q-S laser-assisted tattoo removal represents another interesting approach to quickly release Q-S laser-induced gasses trapped within sub-epidermal bubbles [13–15]. Besides providing an easy way to quickly eliminate intradermal gasses, this approach also contributes to partially eliminate intradermal pigments encountered along the vertical micro-columnar dermal perforations, irrespective of their depths and color. Minimal external elimination of tattoo pigments could also be possible during the first one to two post-treatment days when regular dressing changes are performed. Sequential combinations of ablative fractional 2940-nm Er:YAG laser and 2–3 passes of full-beam Q-S 1064-nm Nd:YAG laser have already proven to be quite effective in eliminating tattoo pigments [8, 16] (see Fig. 1). As for the aforementioned multi-pass laser-assisted intradermal pigment removal techniques, even this innovative approach bears some disadvantages, the most relevant being the need to operate with two separate laser devices, a scenario not always available in most dermatological practices. With the advent of more powerful Q-S laser systems and sophisticated fractional optics, the versatile 1064-nm Nd:YAG wavelength has been used to produce highly efficient micro-ablative photo-acoustic tissue “drilling,” reaching depths comparable to those produced by common fractional ablative lasers. Almost immediate pinpoint capillary bleeding, quite similar to that observed after 2940-nm Er:YAG and 10.600-nm CO2 fractional ablative lasers, is clinically evident after fractional ablative Q-S 1064-nm laser, confirming micro-ablative penetrations down to papillary and superficial reticular vascular plexuses.

The aim of this case series was to assess safety and efficacy of a sequential combination of a Q-S 1064-nm laser photo-acoustic fractional ablative skin “priming” followed by two full-beam 1064-nm Q-S laser passes, spaced 5 min apart, to remove intradermal tattoo pigments.

## Materials and methods

### Study population

Ten healthy Fitzpatrick skin types II patients (7 females and 3 males) between 18 and 57 years of age (mean 28) were enrolled in this study. A total of 13 tattoos, mostly monochromatic, were identified and selected for treatment. All tattoos were reported to be asymptomatic and had never been treated before. Anatomical distribution was different, with the majority of tattoos — seven lesions — located on the upper body, with six on the upper extremities and one on the neck. Ten out of the thirteen tattoos were characterized by variable hues of intradermal black ink. The remaining three tattoos showed a combination of black color with green, red, and pink/purple hues.

The study has been performed in accordance with the Declaration of Helsinki, and all patients signed a specific informed consent prior to each laser session.

### Laser treatment protocol

Local field block infiltration anesthesia (2% mepivacaine + 1:200,000 epinephrine) was administered on all study subjects 5–10 min before each sessions to optimize patients’ comfort during and immediately after laser procedures. Epinephrine-induced capillary vasoconstriction temporary reduces dermal hemoglobin concentration, lowering its potential interference with optical penetration of 1064-nm and 532-nm laser beams. All tattoos were treated with the same Q-S laser system (StarWalker MaQX, Fotona, Slovenia), using 1064-nm wavelength to target the majority of dark tattoo pigments and 532 nm to address the sparingly distributed red colors. The specific pulse produced by this laser system (MaQX pulse) consists of a burst of high peak power 500–600-ps micro-pulses delivered in a 5-ns super-pulse. A standardized multi-pass laser technique, consisting of three subsequent laser passes separated by 5-min intervals, was performed on all tattoos. The first pass consisted of a 1064-nm Q-S laser used in fractional photo-acoustic micro-ablative mode delivered through a 9 × 9 mm focused handpiece. This pass was able to drill a precise array of 81 micro-channels, 0.2 mm in diameter, regularly spaced within precise square 9 mm × 9 mm spots. A standardized laser fluence of 86 mJ/px was used producing almost immediate pin-point capillary bleeding (Fig. 3). Inter-pass temporary dressing, consisting of wet sterile gauze soaked in cold (4 °C) 0.9% normal saline solution, covered by soft 1–2 °C chilled conforming gel pads, was used during the 5-min intervals separating each laser passes. This simple strategy was able to effectively stop laser-induced capillary bleeding. Gentle cleansing to eliminate coagulated superficial blood residues and micro-tissue debris allowed a perfect visual perception of tattoo designs. Most tattoos received two subsequent full-beam 1064-nm Q-S laser passes — always spaced 5-min apart — using a 4.0-mm collimated handpiece with fluences ranging from 5.6 to 9.4 J/cm^2^. Fluence selection varied according to density and distribution of tattoo pigments; higher pigment concentrations required lower fluences. Red pigments were treated with 532-nm Q-S laser passes using a 4-mm collimated full-beam handpiece with fluences ranging from 4.0 to 4.2 J/cm^2^. When needed, Q-S 532-nm laser passes were performed immediately after each Q-S 1064-nm full-beam laser pass. Laser beams were always oriented perpendicularly to skin surface with a minimum of 10% overlap. A lower intensity (6–6.8 J/cm^2^) Q-S 1064-nm laser pass using a 4-mm collimated full-beam laser handpiece was performed to fade the borders of treated tattoos with the aim of reducing peripheral demarcation lines potentially associated with the so-called tattoo ghost effect.

### Post-laser skin care

A standardized post-procedure dressing sequence, consisting of sterile paraffin gauze followed by a thin layer of alginate gel (Flaminal Forte, Flen-Pharma, Belgium) and sterile viscose compresses (Metalline, Lohmann & Raucher, Germany), was performed over each tattoos. Dressings were stabilized by hypoallergenic surgical tape (Hypafix, BSN Medical, Germany). Once discharged, all patients were instructed to change the same dressing sequence twice a day until complete re-epithelization was achieved. Gentle skin cleansing with a delicate product (Neo-Cyteal, Pierre Fabre Dermatologie, France), and topical application of 1.5% H_2_O_2_ to remove skin debris was prescribed before each dressing change. A once-a-day application of a sterile, flexible hydrocolloid dressing (Suprasorb H extra-thin, Lohmann & Rauscher, Germany) was prescribed once epidermal healing was achieved and continued for a total of 7–10 more days.

The third post-procedure skin care step consisted of topical applications of a super-potent (class 3) steroid ointment (0.05% clobetasol propionate) twice a day for 2–3 consecutive days per week — with the aim of controlling excessive localized reactive neo-angiogenesis and preventing post-inflammatory hyperpigmentation. This “pulsed” topical steroid therapy was continued for 1–2 months. A topical 50 + SPF moisturizing cream (Cicaplast baume B5, La Roche Posay, France) was prescribed to be liberally applied 2–3 times a day during the same period, and continued until subsequent laser sessions.

Laser sessions were performed, at 2–3-month average intervals, until clearance was achieved.

### Clinical evaluation and data analysis

**Fig. 2:**
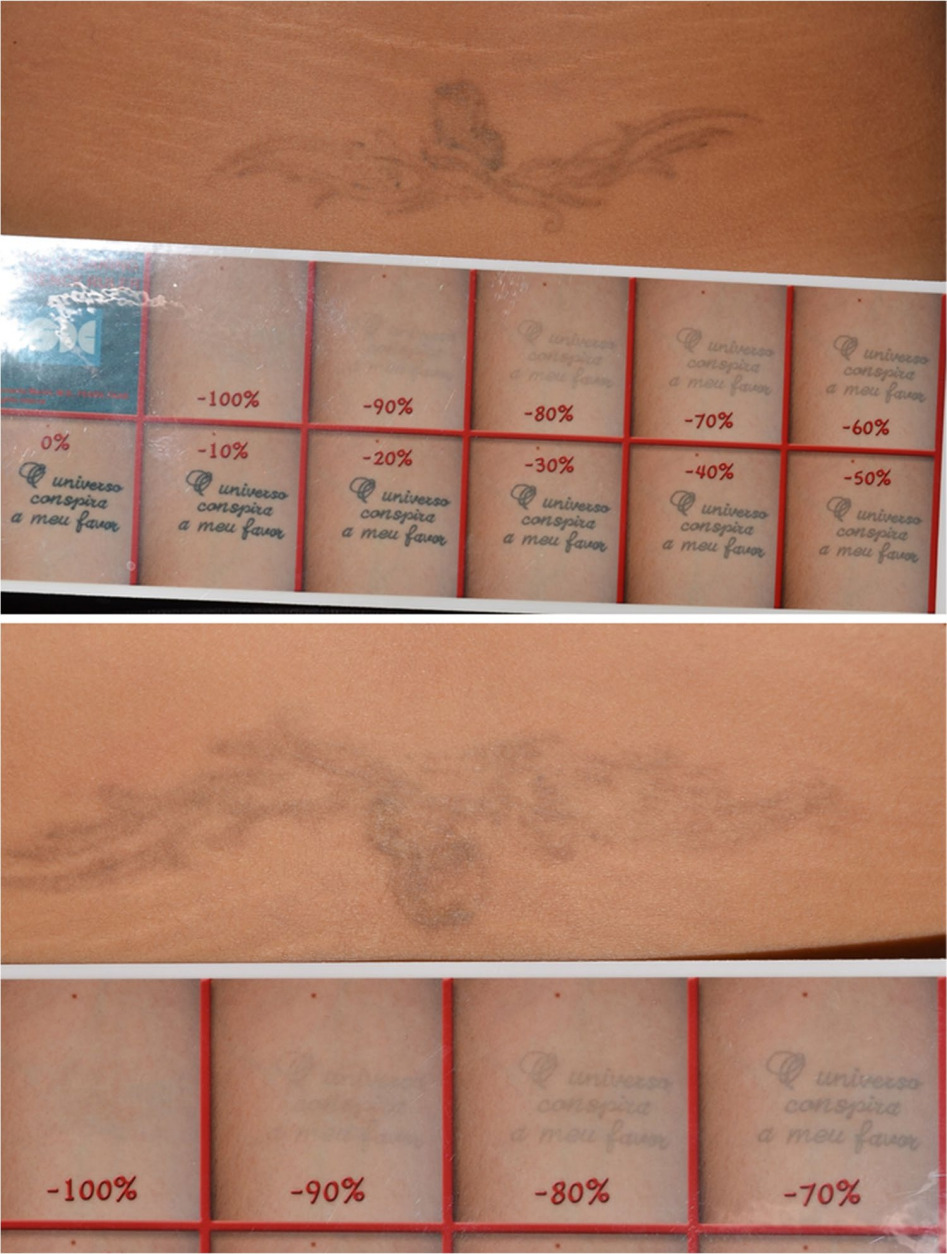
The custom-made pigment-fading percentage ruler for objectively assessing tattoo pigment clearance efficiency developed at the Skin Doctor Center – Trieste (I). The ruler consists of a series of 11 digitally modified photographs showing progressive 10% color saturation decrements between two extremities: 0% (intact virgin tattoo) and 100% (complete pigment removal)

The estimated number of laser sessions to obtain a complete pigment clearing using a single Q-S laser PPS, according to the Kirby-Desai scale [17], was discussed and recorded for each tattoo during the first clinical evaluation. A Canon EOS 400D digital camera equipped with a Canon 50-mm macro-lens and a dedicated Canon 270EXII TTL flash was used to collect all clinical images. Two digital images were taken immediately before each laser session for each tattoo. The first picture framed the whole tattoo. The second picture included a custom-made pigment-fading photographic ruler, specifically developed in our Clinic. Ruler was positioned at the immediate periphery of each tattoo (Fig. 1). This ruler consists of a consecutive series of eleven progressively fading clinical pictures originating from a fully saturated black tattoo image, arbitrarily chosen as reference from our image files. Pigment saturation goes from 0% of the original tattoo image to 100% of digitally modified complete pigment cleared picture. The two ends of our ruler are separated by nine digitally modified Photoshop pictures featuring progressive 10% color saturation decrements. This simple structured VAS solution proved to be a reliable instrument to assess the effectiveness of laser-assisted tattoo removal efficacy (Fig. 2).

All photographic sequences collected for each tattoo were stored in dedicated files to be finally evaluated by four independent dermatologists, once optimal tattoo pigment clearance was obtained.

The same pictures were used to assess treatment-related side effects such as scarring and pigmentary modifications according to a 0–3 severity scale (0 **—** no pigment changes or scarring; 1 — barely visible pigment changes or scarring, 2 — moderate pigment changes or scarring, and 3 — intense pigment changes or scarring).

The degree of pain/discomfort during laser treatments was assessed by patients according to a 0–10 VAS scale. Overall patient satisfaction was evaluated 2 months after the last laser procedure by all patients according to a 1–5 satisfaction scale, where “1” corresponded to complete dissatisfaction and “5” to total satisfaction.

All results were entered into an anonymized spreadsheet (Microsoft Excel), where descriptive statistical analyses were performed.

## Results

The pigment fading ruler developed in our Clinic proved to be an extremely useful VAS system to assess the efficiency of laser-assisted tattoo removal procedures showing a high inter-evaluator agreement among the four dermatologists involved in the clinical assessment, as confirmed by the low variability in the estimated clearance and side-effect scores. The mean number of sessions to achieve a 90–100% pigment clearance was 4.85. The average number of sessions needed to obtain a good clinical result was 40% lower than that predicted by the Kirby-Desai score based on the single Q-S laser PPS technique. Minimal side effects were associated with our new multi-pass treatment protocol (minimal self-resolving textural changes, transient PIH, and post-treatment erythema). This was confirmed by an average 0.65 score (range 0–1.5) on a 0–3 severity scale. Overall treatment satisfaction perceived by patients was very high (mean 4.8) on a 1–5 satisfaction scale. The mean pain level experienced by the patients during laser sessions was 1.62 (range 1–3) on a 0–10 VAS scale (Table 1).

**Table 1:** Parameters evaluated from 13 tattoos participating to this case series. Kirby-Desai estimates were done prior to treatment. Pigment clearing percentages were estimated by 4 independent dermatologists who were also responsible for the evaluation of side effects. Both values for each patient are presented as a median (range) of those taken by all the evaluators. Pain scores and overall treatment satisfaction were estimated by each patient at the end of each treatment session and once the final clearing results were obtained

Tattoo No	Clearing estimate 4 evaluator median (range)	Side effect score 4 evaluator median (range)	No. of sessions to clearance	Kirby-Desai estimate	Pain during treatment (0–10)	Patient satisfaction (1–5)
1	100% (90–100)	0.5 (0–1)	5	8	2	5
2	100% (100–100)	1 (1–1)	6	8	2	5
3	100% black (100–100) 50% green (20–70)	1 (1–2)	6	8 black 10 green	1	4
4	100% (90–100)	1.5 (0–2)	5	9	2	4
5	100% (90–100)	1.5 (1–2)	4	8	2	5
6	90% (80–90)	1 (1–2)	5	9	3	5
7	100% (100–100)	1 (0–1)	3	7	1	5
8	100% (100–100)	0 (0–0)	7	8	2	5
9	90% (80–90)	0 (0–1)	6	9	1	5
10	100% (100–100)	0 (0–0)	5	6	1	5
11	90% (90–90)	1 (1–2)	5	9	2	5
12	100% (90–100)	0 (0–0)	3	8	1	5
13	100% (100–100)	0 (0–0)	3	7	1	5
∑mean ± SD	97% ± 4	0.65 ± 0.54	4.85 ± 1.19	8	1.62 ± 0.6	4.85 ± 0.35

## Discussion

“Priming” skin with high-energy per pulse Q-S 1064-nm Nd:YAG laser used in a fractional photo-acoustic “micro-drilling” mode was found very useful and effective when combined with two passes of full-beam Q-S 1064-nm Nd:YAG laser, eventually associated with Q-S 532-nm KTP laser when red ink was encountered, in removing intradermal tattoo pigments with minimal side effects. Incidental observations perceived by clinical observers consisted in clinical improvements of skin textural alterations induced by original tattoo procedures, possibly due to tissue remodeling by 1064-nm Q-S fractional micro-ablative passes. All patients participating in this clinical study confirmed a high degree of satisfaction considering the relatively smaller number of sessions needed to achieve an almost complete pigment clearance compared to the estimated number of single-laser PPS calculated according to the Kirbi-Desay score.

Effectiveness and safety of different laser tattoo removal methods have been recently summarized in a systematic review from Gurnani and colleagues [18]. Q-S lasers represent the gold standard technology in tattoo removal. Conventional single PPS treatments, using nanosecond and picosecond-pulse 1064-nm and 532-nm laser wavelengths, have been previously reported as effective and safe methods for tattoo removal. However, when compared to the method reported in our study, both confirmed to have lower clearance rate at comparable numbers of treatment sessions and higher rates of hyperpigmentations/hypopigmentations, the latter being more common with pico-second lasers [18]. Newer approaches aimed at reducing the total number of laser sessions while keeping a good level of pigment clearing include multiple PPS strategies using progressive layering of full-beam Q-S laser, as described by the R-20 technique, or combinations of ablative fractional 2940-nm Er:YAG or CO_2_ laser with one to three passes of full-beam Q-S lasers. We have also previously used a sequential combination of 2940-nm short-pulse Er:YAG ablative fractional micro-drilling followed by two full-beam 1064-nm and 532-nm passes producing excellent clinical results, comparable to those observed in this study [8, 15]. However, the need to work with two different laser systems constitutes a relative disadvantage, both in terms of economical investment and ergonomic use of space within treatment rooms.

For this study we decided to use a single, latest generation high-power Q-S laser device (StarWalker, Fotona, Slovenia) offering the option of 1064-nm Q-S photo-acoustic fractional micro-ablative mode to reach intradermal depths, as confirmed by clinical observations featuring almost immediate pinpoint bleeding, as well as full beam collimated large spot (> 4 mm) high power density mode. Preliminary tissue micro-drilling generating micro-channels perpendicular to skin surface allows elimination of all pigment particles distributed along micro-columnar laser paths, irrespective of their colors and depths. Skin micro-perforation allows gas bubbles induced by full-beam 1064-nm Q-S laser passes interacting with dermal tattoo pigments to escape outside, significantly decreasing waiting time between laser passes when multiple PPS strategies are used [15]. Micro-structural skin improvements are possible when lasers are used in fractional ablative and non-ablative modes as widely demonstrated in scar and skin aging treatments. Q-S 1064-nm micro-drilling seems to produce the same clinical effects.

**Fig. 3:**
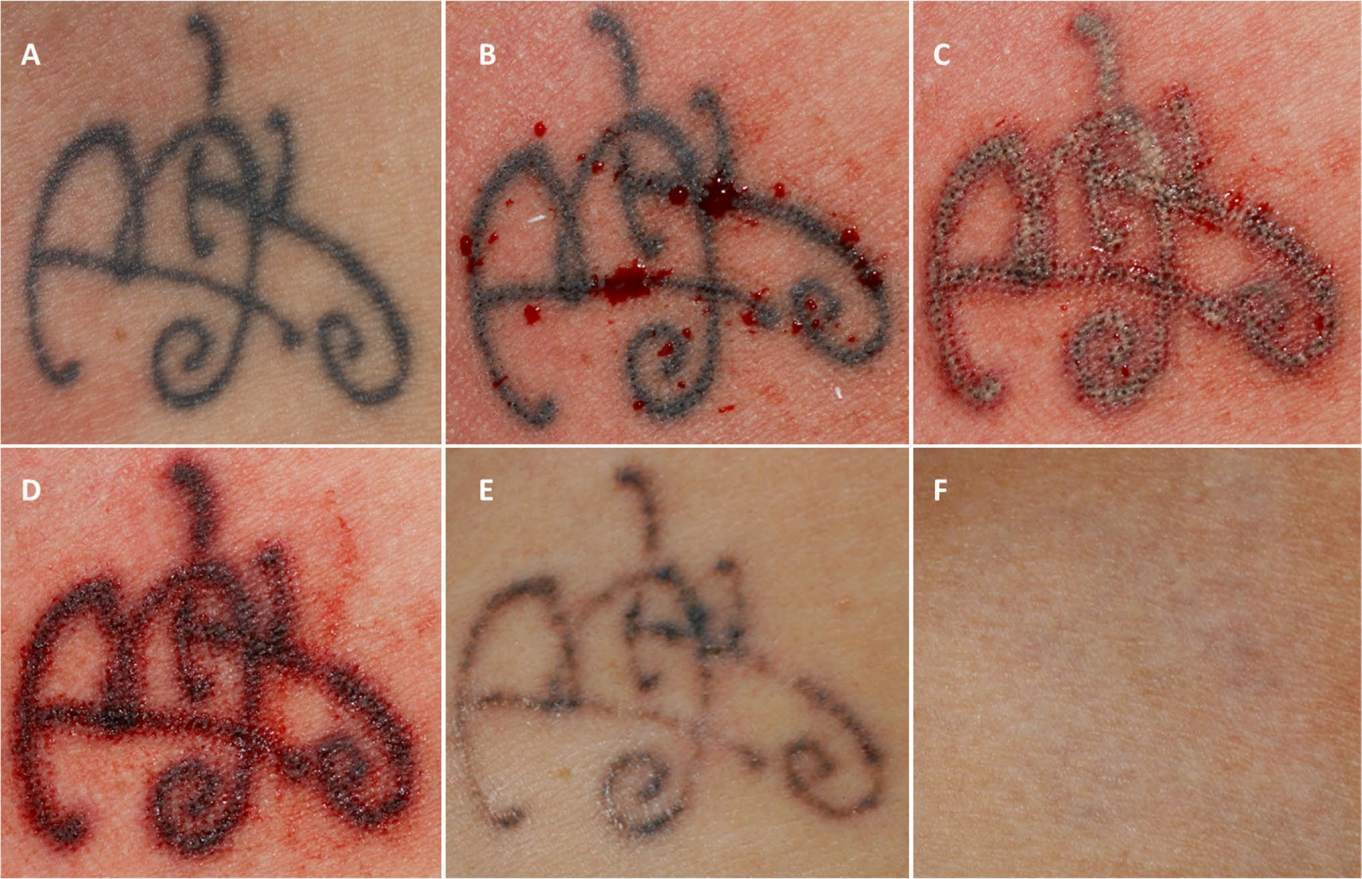
Professional monochromatic black tattoo located on the upper chest of a 30-year-old female patient. **A** Before the laser procedure; **B** immediately after 1064-nm Q-S micro-drilling — pin-point capillary bleeding clearly visible; **C** immediately after the first full beam Q-S 1064-nm laser pass — note the fragmented, whitish sub-epidermal discoloration representing the Q-S micro-drilling modified pop-corn effect; **D** immediately after the second Q-S 1064-nm full-beam laser pass; **E** clinical result 1 month after the first laser session; **F** complete tattoo clearance after four laser sessions — a 100% pigment clearance score and side effect score of 1.5 were confirmed by all dermatologists asked to evaluate the case

**Fig. 4:**
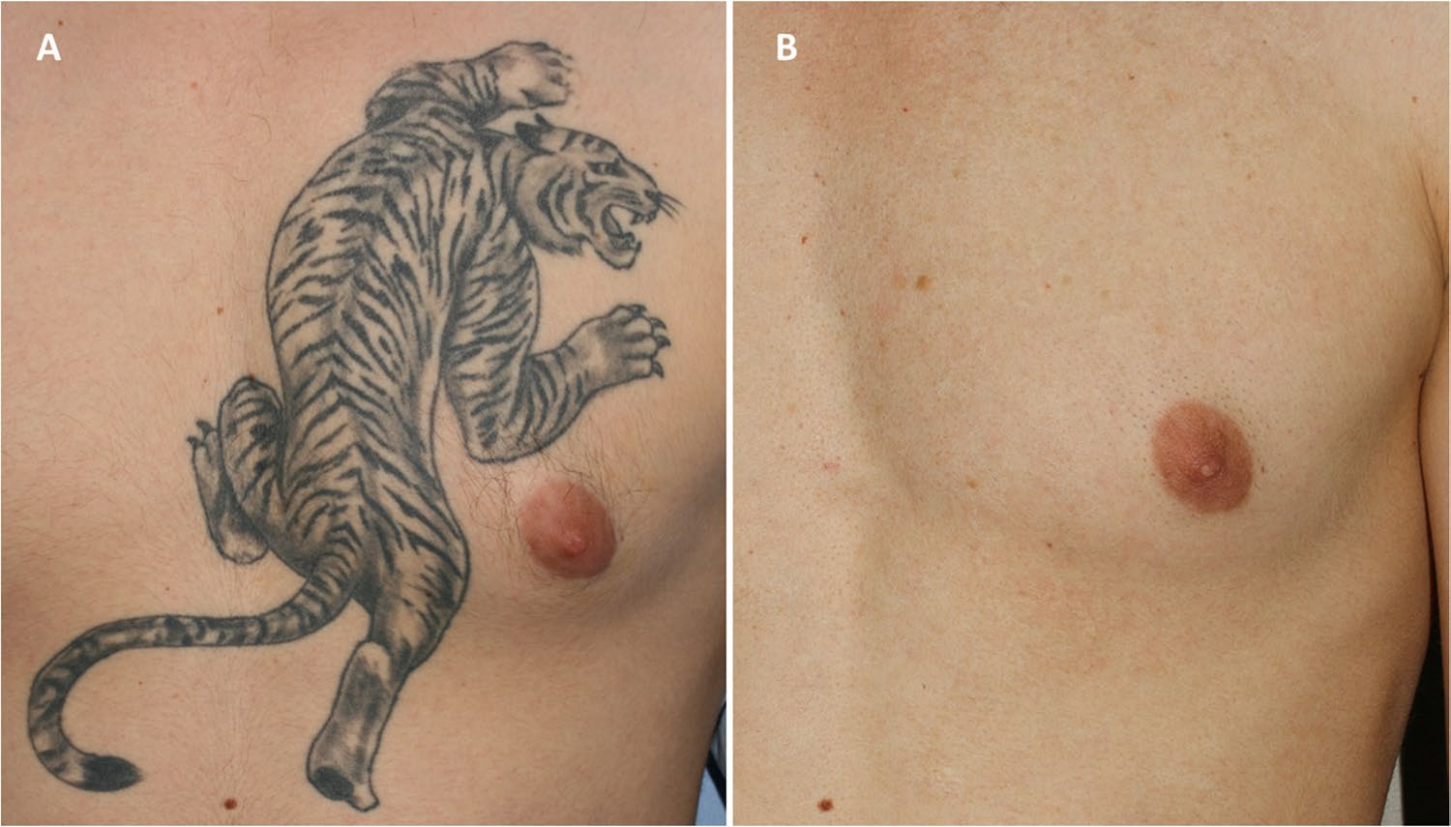
Professional monochromatic black tattoo on the anterior chest of a 29-year-old male patient. **A** Before treatment; **B** clinical result after seven laser sessions — 100% pigment clearance score and a side effect score 0 were agreed by all four dermatologists responsible for evaluating the case

**Fig. 5:**
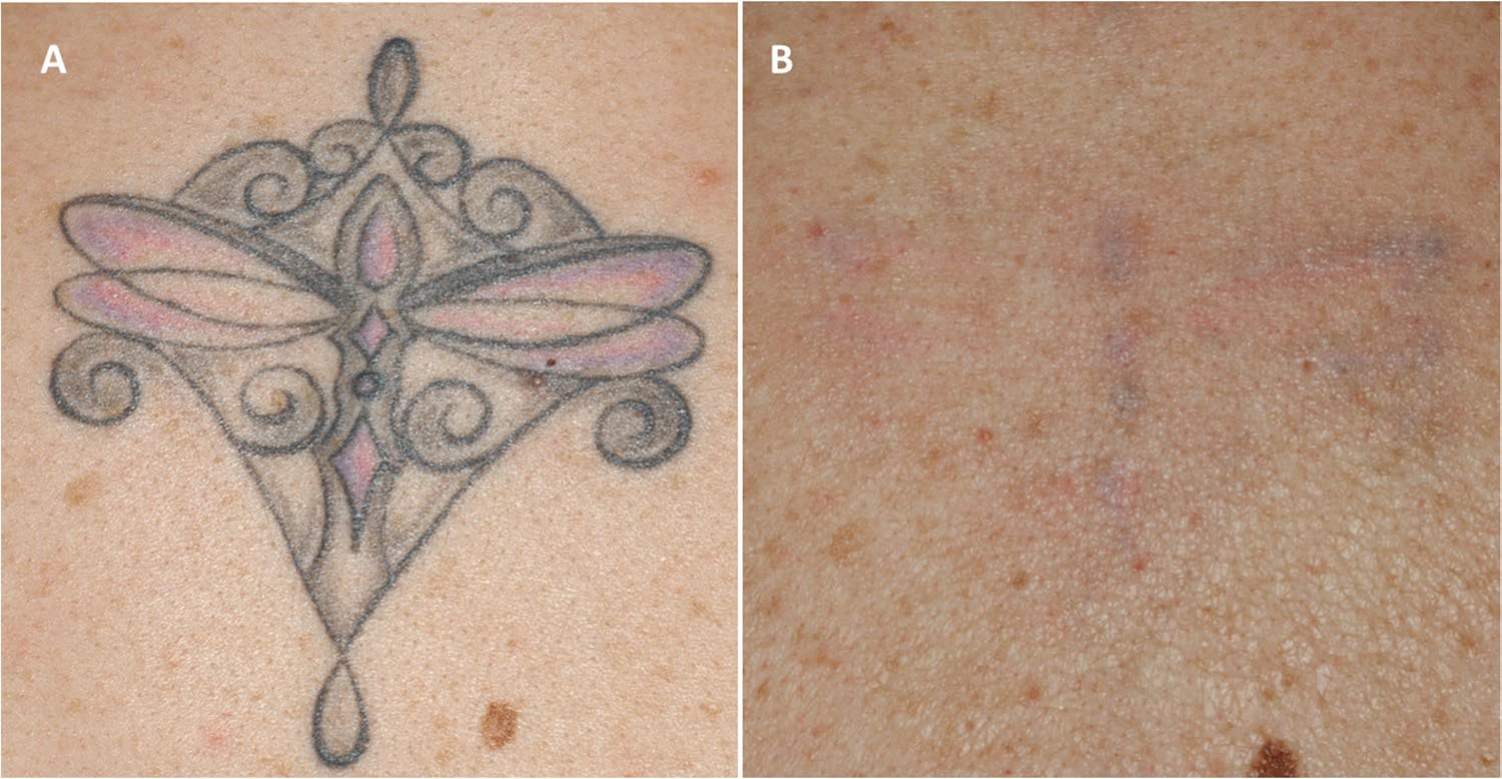
Professional polychromatic black-pink-purple tattoo on the median upper dorsal region of a 40-year-old female patient. **A** Before treatment; **B** clinical result after 6 treatment sessions — 90% pigment clearance and 0 side effect score (median values confirmed by expert evaluators)

**Fig. 6:**
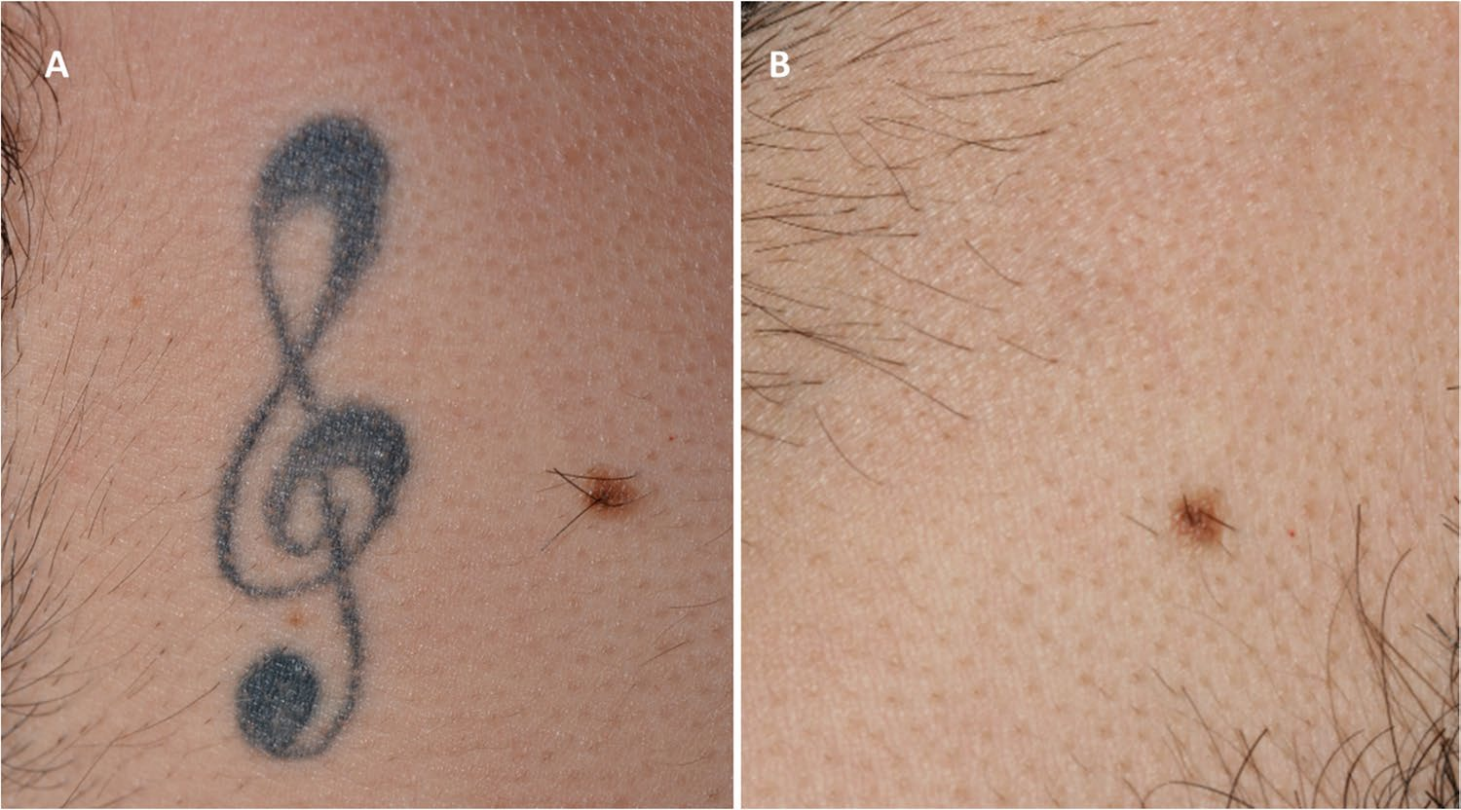
Professional monochromatic black tattoo on the upper neck of a 27-year-old male patient. **A** Before laser treatment; **B** complete clearance after five treatment sessions; pigment clearance was estimated to be 100%, with a side effect score of 0 by all four dermatologists asked to evaluate the case

One of the most significant advantages associated with the multi-pass laser-assisted tattoo removal strategy described in our study resides in the fact that all its sequential steps can be performed using a single high-powered Q-S laser system. The average number of sessions to obtain an almost complete pigment clearance was 4.85, with 4 sessions needed to get a 90% or greater clearance, on average. The mean number of sessions compared to the classical single Q-S laser PPS protocol, as determined by the Kirby-Desai score, was reduced by 40%. Besides that, scarring, erythema, and pigmentary changes showed to be minimal. Skin texture was significantly improved due to tissue remodeling effects induced by high energy 1064-nm Q-S fractional micro-drilling pass (Figs. 3, 4, 5, and 6).

In spite of these positive clinical achievements, it is always wise to remember that any laser procedure is not over when laser systems are switched off. All procedures require a specifically designed post-treatment skin care to obtain an optimal, fast healing. This laser method is no different. Without the suggested post-treatment dressing sequence, healing time and quality of tissue repair would have been quite different.

High-power, short-pulse Q-S nanosecond lasers have been proven to work almost equally well as picosecond Q-S lasers, even if these systems have gained quite a spectacular popularity in recent years [19]. In addition, most picosecond Q-S lasers available on the market to date are not able to produce high-enough energy outputs to produce the FracTat® micro-drilling effect described in our study.

## Conclusions

Despite important advances in laser-assisted tattoo removal techniques over the past decade, no general agreement yet exists about what should be considered the safest and most effective approach. The combined approach described in this study demonstrated comparable results to previously described Q-S laser strategies in terms of final exogenous pigment fading, but with a greater efficiency in pigment removal, significantly decreasing the total number of sessions required to obtain a good clinical result. The use of a latest-generation, high power Q-S laser system allowed an efficient photo-acoustic fractional ablation of skin layers as deep as reticular dermis when 1064-nm wavelength was selected. The sequential combination of preliminary 1064-nm Q-S ablative micro-drilling and two full-beam 1064-nm Q-S laser passes showed to be safe and effective in tattoo pigment removal. The weak point of our study is the relatively small number of treated lesions. Larger numbers will be necessary to further confirm our findings.
