# Correction to: Q‑S laser micro‑drilling and multipass full‑beam Q‑S laser for tattoo removal — a case series

**DOI:** 10.1007/s10103-021-03468-x

**Published:** 2021-12-02

**Authors:** Leonardo Marini, Susanna Marini, James Cutlan, Irena Hreljac

**Affiliations:** 1 SDC The Skin Doctors Center, via dei Bonomo 5/a, 34126 Trieste, Italy; 2 University Hospital of Wales, Cardiff, Wales UK; 3 Royal Glamorgan Hospital, Llantrisant, Wales UK; 4 Laser and Health Academy, Ljubljana, Slovenia

**Correction to: Lasers Med Sci**

**https://doi.org/10.1007/s10103-021-03431-w**

The article “Q‑S laser micro‑drilling and multipass full‑beam Q‑S laser for tattoo removal — a case series”, written by Leonardo Marini, Susanna Marini, James Cutlan and Irena Hreljac, was originally published electronically without open access. With the author(s)’ decision to opt for Open Choice the copyright of the article changed on 11 November 2021 to © The Author(s) 2021 and the article is forthwith distributed under a Creative Commons Attribution 4.0 International License, which permits use, sharing, adaptation, distribution and reproduction in any medium or format, as long as you give appropriate credit to the original author(s) and the source, provide a link to the Creative Commons licence, and indicate if changes were made. The images or other third party material in this article are included in the article’s Creative Commons licence, unless indicated otherwise in a credit line to the material. If material is not included in the article’s Creative Commons licence and your intended use is not permitted by statutory regulation or exceeds the permitted use, you will need to obtain permission directly from the copyright holder. To view a copy of this licence, visit http://creativecommons.org/licenses/by/4.0.

The original article has been corrected.

